# Fraction of *C. d. collilineatus* venom containing crotapotin protects PC12 cells against MPP ^+^ toxicity by activating the NGF-signaling pathway

**DOI:** 10.1590/1678-9199-JVATITD-2023-0056

**Published:** 2024-06-14

**Authors:** Carolina Petri Bernardes, Ernesto Lopes Pinheiro, Isabela Gobbo Ferreira, Isadora Sousa de Oliveira, Neife Aparecida Guinaim dos Santos, Suely Vilela Sampaio, Eliane Candiani Arantes, Antonio Cardozo dos Santos

**Affiliations:** 1 Department of Clinical Analyses, Toxicology and Food Science, School of Pharmaceutical Sciences of Ribeirão Preto, University of São Paulo (USP), Ribeirão Preto, SP, Brazil.; 2 Department of Biomolecular Sciences, School of Pharmaceutical Sciences of Ribeirão Preto, University of São Paulo (USP), Ribeirão Preto, SP, Brazil.

**Keywords:** Crotapotin, Snake venom, Neuroprotection, Neuritogenesis, MPP^+^, Parkinson's disease

## Abstract

**Background:**

Parkinson’s disease (PD) is the second most prevalent neurodegenerative disease. There is no effective treatment for neurodegenerative diseases. Snake venoms are a cocktail of proteins and peptides with great therapeutic potential and might be useful in the treatment of neurodegenerative diseases. Crotapotin is the acid chain of crotoxin, the major component of *Crotalus durissus collilineatus* venom. PD is characterized by low levels of neurotrophins, and synaptic and axonal degeneration; therefore, neurotrophic compounds might delay the progression of PD. The neurotrophic potential of crotapotin has not been studied yet.

**Methods:**

We evaluated the neurotrophic potential of crotapotin in untreated PC12 cells, by assessing the induction of neurite outgrowth. The activation of the NGF signaling pathway was investigated through pharmacological inhibition of its main modulators. Additionally, its neuroprotective and neurorestorative effects were evaluated by assessing neurite outgrowth and cell viability in PC12 cells treated with the dopaminergic neurotoxin MPP^+^ (1-methyl-4-phenylpyridinium), known to induce Parkinsonism in humans and animal models.

**Results:**

Crotapotin induced neuritogenesis in PC12 cells through the NGF-signaling pathway, more specifically, by activating the NGF-selective receptor trkA, and the PI3K/Akt and the MAPK/ERK cascades, which are involved in neuronal survival and differentiation. In addition, crotapotin had no cytotoxic effect and protected PC12 cells against the inhibitory effects of MPP^+^ on cell viability and differentiation.

**Conclusion:**

These findings show, for the first time, that crotapotin has neurotrophic/neuroprotective/neurorestorative potential and might be beneficial in Parkinson's disease. Additional studies are necessary to evaluate the toxicity of crotapotin in other cell models.

## Background

Venoms are complex and specialized mixtures of enzymatic and non-enzymatic proteins, peptides, and non-protein compounds with great pharmacological potential [1-4]. These bioactive molecules target ion channels, receptors, and a variety of modulators. They might serve as the basis for developing new drugs for treating several diseases, including neurodegenerative diseases [5].

*Crotalus durissus collilineatus* (*C. d. collilineatus*) is a subspecies found in the Brazilian Southeast and Central West regions [6, 7]. Its venom consists of different classes of proteins and peptides like bradykinin-potentiating peptides, convulxin, crotamine, crotoxin, and gyroxin [7-10], crotoxin being the major component [11-13]. Crotoxin is a molecule composed of two subunits that are non-covalently bonded, i.e., subunit A or crotapotin (crotoxin acid chain) and subunit B or phospholipase A_2_ (PLA_2_) [11, 14-17]. Crotapotin is a non-toxic, non-enzymatic protein [6, 14] with anti-inflammatory [18-20], and antimicrobial activities [16, 18, 19, 21, 22]. The neurotrophic activity of crotapotin is unknown. This study investigates if crotapotin has neurotrophic potential and if it could be involved in the neuroprotection against the toxicity induced by MPP^+^. This neurotoxin is the active metabolite of MPTP (1-methyl-4-phenyl-1,2,3,6-tetrahydropyridine). Both MPTP (parent drug) and MPP+ (its active metabolite) are associated with Parkinsonian syndrome in primates [23].

Parkinson's disease (PD) is the second most prevalent neurodegenerative disease; it is characterized by synaptic and axonal degeneration, loss of dopaminergic neurons, and reduced levels of dopamine in the nigrostriatal pathway. The treatment of PD is restricted to motor symptoms’ alleviation, without any beneficial effect on cognitive decline. Moreover, long-term treatment induces important side effects and adaptive tolerance [24-26].

Neuronal survival, differentiation, and regeneration are controlled by neurotrophins both during the nervous system development and in the mature nervous system [27-30]. Studies provide evidence that reduced levels of neurotrophins are involved in the pathogenesis of neurodegenerative diseases [31-35]. Therefore, compounds that mimic or enhance the action of neurotrophins might be of use to slow the progression of neurodegeneration or restore the lost neuronal function [36-38]. In this scenario, animal toxins represent a promising source of new molecules with neuroprotective activity and therapeutic potential against neurodegeneration [39-43]. 

Based on these premises, this study investigates the neuroprotective activity of crotapotin in PC12 cells treated with the dopaminergic neurotoxin MPP^+^, with a focus on the neurotrophic signaling pathway triggered by the neurotrophin NGF as a possible mechanism of neuroprotection. This is a prospective work in which different isoforms of crotapotin have been tested for the initial characterization of the neurotrophic and neuroprotective effects of crotapotin.

## Methods

### Reagents

1-Methyl-4-phenylpyridinium iodide (MPP+, D048), LY-294002 hydrochloride (L9908), 3-(4,5-Dimethyl-2-thiazolyl)-2,5-diphenyl-2H-tetrazolium bromide (MTT, M2128), K252a (K2015), U0126 monoethanolate, 4-(2-hydroxyethyl)-1-piperazineethanesulfonic acid (HEPES, H3375), sodium bicarbonate (S5761), Dulbecco's Modified Eagle Medium (DMEM) (D5648), Ham′s F12K medium (N6658), Trypsin-EDTA solution (59427C), Nerve Growth Factor (NGF) from *Vipera lebetina* venom (N8133), Collagen Type-IV (C5533) and Fetal bovine serum (F2561) were purchased from Sigma-Aldrich (USA). Equine serum and antibiotic mix (PSN, 5 mg/mL penicillin, 5 mg/mL streptomycin, and 10 mg/mL neomycin) were purchased from GIBCO^®^ (Life Technologies Corporation, USA). 

### Crotapotin identification

*C. d. collilineatus* venom fractionation and crotapotin isolation were performed as described previously [44]. Crotapotin purity was assessed by Fast Protein Liquid Chromatography or FPLC (Äkta Purifier UPC10 GE Healthcare, Sweden), with a reversed-phase C4 Jupiter column (4.6 × 250 mm, 5 μm, 300 Å, Phenomenex, USA), employing 0.1% trifluoroacetic acid (TFA) as solution A, and 80% acetonitrile (ACN) + 0.1% TFA as solution B. The elution was performed through a linear gradient of 0-100% solution B, at a 1 mL/min flow rate. Absorbance was monitored at 280 nm.

Additionally, crotapotin was analyzed by matrix-assisted laser desorption/ionization (MALDI) with a time of flight (TOF) analyzer to determine its molecular mass and identity. The molecular mass was analyzed by using an ultrafleXtreme instrument (Bruker Daltonics GmbH, Leipzig, Germany) with the Smartbeam II laser. Data acquisition was performed with FlexControl software, version 3.3 (Bruker Daltonics GmbH, Leipzig, Germany). The following parameters were employed: 500 laser shots per spectrum; 1000 Hz laser frequency; positive reflected mode; and a range of 5 to 30 kDa. A mixture of peptides (Peptide calibration standard, NC9846988, Bruker Daltonics GmbH, Leipzig, Germany) and proteins (Protein calibration standard I, NC0239984, and Protein calibration standard II, NC0416074, Bruker Daltonics GmbH, Leipzig, Germany) was used for calibration. As a matrix, a saturated solution of α-cyano-4-hydroxycinnamic acid (α-CHCA) in ACN and 0.1% TFA (V/V), at the ratio of 1:1 (V/V) was used. The software FlexAnalysis, version 3.3 (Bruker Daltonics GmbH, Leipzig, Germany) was used for data analysis.

For identification, the crotapotin fraction that eluted from RP-FPLC was reduced, alkylated, digested with sequencing grade porcine pancreatic trypsin, and analyzed by Axima Performance (Shimadzu, Manchester, UK). MS/MS data were analyzed with the MASCOT program, against databank protein sequence deposited in the NCBI (65,519,838 sequences, 23,472,502,492 residues) and SwissProt (548,208 sequences, 195,282,524 residues). Cysteine carbamidomethylation was included as a fixed modification, and methionine oxidation was included as a variable modification. MS/MS mass tolerance was set to ± 0.8 Da.

### Cell culture

Rat pheochromocytoma PC12 cell line (PC12 - CRL-1721) was obtained from the American Type Culture Collection (ATCC, USA). PC12 cells were cultured in high-glucose DMEM, supplemented with 10% equine serum, 5% fetal bovine serum, and 1% antibiotic mix, at 37 °C in a humidified atmosphere containing 5% CO_2_. The medium was replaced every 2 days. For the assays, cells were detached with trypsin-EDTA solution and the enzymatic reaction was stopped with an equal volume of culture medium.

### Differentiation/neurite outgrowth assay

PC12 cells (2 × 10^5^ cells/well) were treated with crotapotin (2.5; 5; and 10 µg/mL) or NGF (100 ng/mL, positive control) and incubated for 72 h. Untreated cells were used as negative controls. Three independent experiments from different cell cultures were assayed; each sample was assayed in triplicate. The morphometric analysis was performed under inverted phase contrast microscopy (Carl Zeiss Axio Observer A1 Inverted Microscope, magnification of 400×). The wells were scanned from left to right and three fields/wells were randomly selected; investigators were blinded about cell treatments (numbers identified groups). The percentage of cells with neurites was determined in digitalized images by using the ImageJ open source software [45]. Cells bearing at least one neurite longer than the diameter of their cell bodies were considered neurite-bearing cells [46]. Data were expressed as a percentage of total cells. 

### Inhibition of NGF-signaling pathway

PC12 cells (2.0 × 10^5^ cells/well) were grown in DMEM supplemented with 10% equine serum, 5% fetal bovine serum, and 1% antibiotic mix. After 24 hours, the medium was replaced with F-12K Nutrient Mixture Kaighn's Modification (GIBCO®, Life Technologies Corporation, USA) supplemented with 1% equine serum and 1% antibiotic mix (PSN, GIBCO®). Crotapotin-induced cell differentiation was evaluated in the presence of (i) the antagonist of trkA receptor (K252a), or the inhibitors of the (ii) PI3K/Akt pathway (LY294002) or (iii) MAPK/ERK pathway (U0126). PC12 cells were pretreated with K252a (100 nM), LY294002 (30 µM), or U0126 (10 µM) as previously described [47, 48] with minor modifications, and incubated for one hour prior to the addition of crotapotin (10 µg/mL) or NGF (100 ng/mL), following incubation for 72h. Neurite outgrowth assay was performed as described for the Neurite Outgrowth Assay.

### Evaluation of the protective effects of crotapotin against MPP-induced toxicity on neuritogenesis

PC12 cells were plated onto 24 well plates (2 × 10^5^ cells/well) and incubated. After 24h, cells were treated with crotapotin (10 µg/mL) and/or MPP^+^ (100 μM). Cells treated with NGF were used as positive controls and untreated cells were used as negative controls. This concentration of MPP^+^ (100 μM) inhibits neurite outgrowth without inducing cell death, as we have previously determined [49]. Neurite outgrowth assay was performed as described in the section “Differentiation/Neurite outgrowth assay”. 

### Evaluation of the protective effects of crotapotin against MPP-induced toxicity on cell viability 

Cells (2 × 10^4^ cells per well, final volume of 200 μL) were plated onto 96-well plates. After 24 hours of incubation, cells were treated with crotapotin (10 µg/mL) and/or MPP^+^ (1 mM). This concentration of MPP^+^ (1 mM) corresponds to the IC_50_ for viability in PC12 cells, as we have previously determined [49]. After 72 hours of treatment, MTT solution (5 mg/mL, 20 μL/well) was added and the plates were incubated (3h, 37 °C). Then, the plates were centrifuged (1000 rpm, 5 minutes), the supernatant was removed and the crystals formed were solubilized in DMSO (200 μL/well). Untreated cells were used as negative controls and cells treated with Triton X-100 were used as positive controls. Samples were assayed in triplicate. The plates were mixed (37 °C, 5 minutes) and the absorbance was determined at 570 nm, in a microplate reader (Multiskan™ FC Microplate Photometer, Thermo Scientific, USA). This procedure was based on previous reports [50, 51].

### Statistical analysis

All data were expressed as mean ± SEM (standard error of the mean). Multiple comparisons were performed by the One-way ANOVA test and Post-hoc Tukey’s multicomparison test (GraphPad Software, San Diego, CA, USA). Values of p < 0.05 were considered significantly different.

## Results

### Fractionation of venom and isolation of crotapotin 

Crotapotin homogeneity, mass spectrometry, and protein sequencing are presented as Supplementary Material (Additional file 1A-1C). 

As previously reported, the fractionation of *Crotalus durissus collilineatus* venom yielded six fractions containing crotapotin, corresponding to the subunit A of the crotoxin complex [44]. There are several isoforms of the subunits A and B of crotoxin with different biological properties [8, 11, 12, 52-56]. In the present study, we evaluated six fractions of crotapotin for their ability to induce differentiation in PC12 cells (Additional file 2A-2B); the most effective, with the lower degree of contaminants (fraction 4) was selected to perform the mechanistic assays and the neuroprotection evaluation.

### Crotapotin-induced cellular differentiation in NGF-deprived PC12 cells 

**Figure 1.**
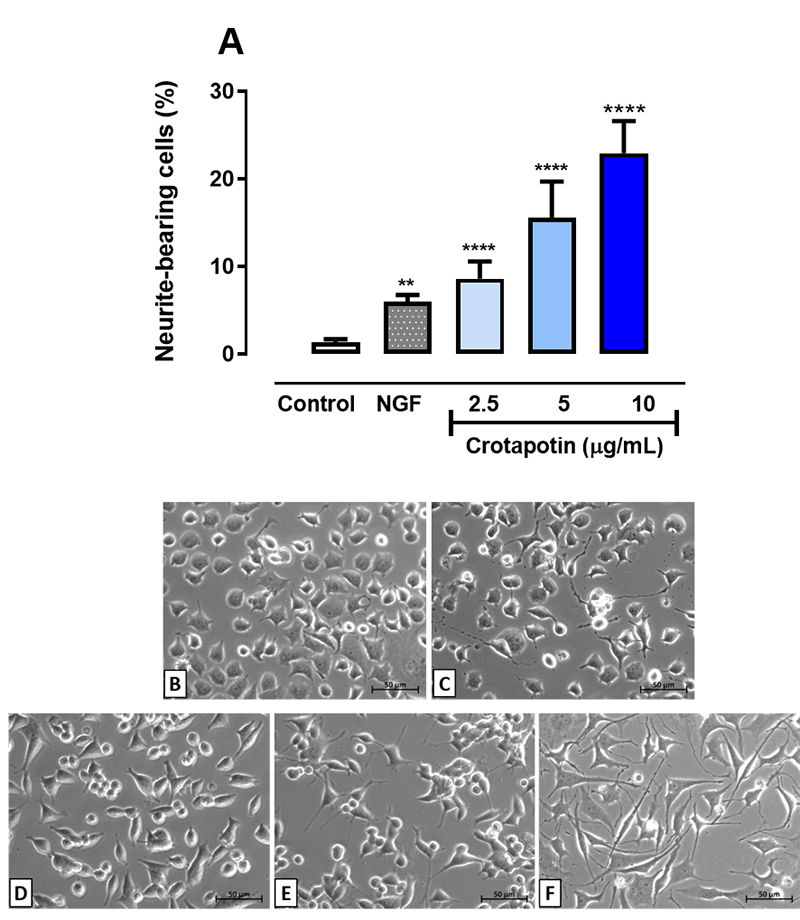
Effects of different concentrations of crotapotin on the differentiation of PC12 cells after 72h incubation. **(A)** The bar graph represents the mean ± SEM (n = 3). Cells with at least one neurite with a length equal to or greater than the cell body were considered differentiated and expressed as a percentage of total cells in the field. **(B-F)** Inverted phase-contrast photomicrographs of **(B)** control (untreated), **(C)** NGF (100 ng/mL), **(D)** crotapotin (2.5 µg/mL), **(E)** crotapotin (5 µg/mL) and **(F)** crotapotin (10 µg/mL). Cells with at least one neurite with a length equal to or greater than the cell body were considered differentiated and expressed as a percentage of the total cells in the field (n = 3). **Significantly different from control (p < 0.01); ****Significantly different from control (p < 0.0001).

Crotapotin significantly induced the differentiation of PC12 cells after 72 hours of incubation when compared to untreated controls (1.31 ± 0.40%). The effect was concentration-dependent as shown by the percentage of neurite-bearing cells stimulated by the following concentrations of crotapotin: 2.5 μg/mL (8.63 ± 1.9%, p < 0.0001), 5 μg/mL (17.21 ± 4%, p < 0.0001) and 10 μg/mL (26.56 ± 3.8%, p < 0.0001). The percentage of neurite-bearing cells in the positive control (NGF-stimulated) was significantly higher (5.96 ± 0.80%, p < 0.01) in comparison with untreated controls (1.31 ± 0.40%). Results are presented in Figure 1 A  -1F.

### Pretreatment with K252a (antagonist of trkA) reduced the differentiation induced by crotapotin

**Figure 2.**
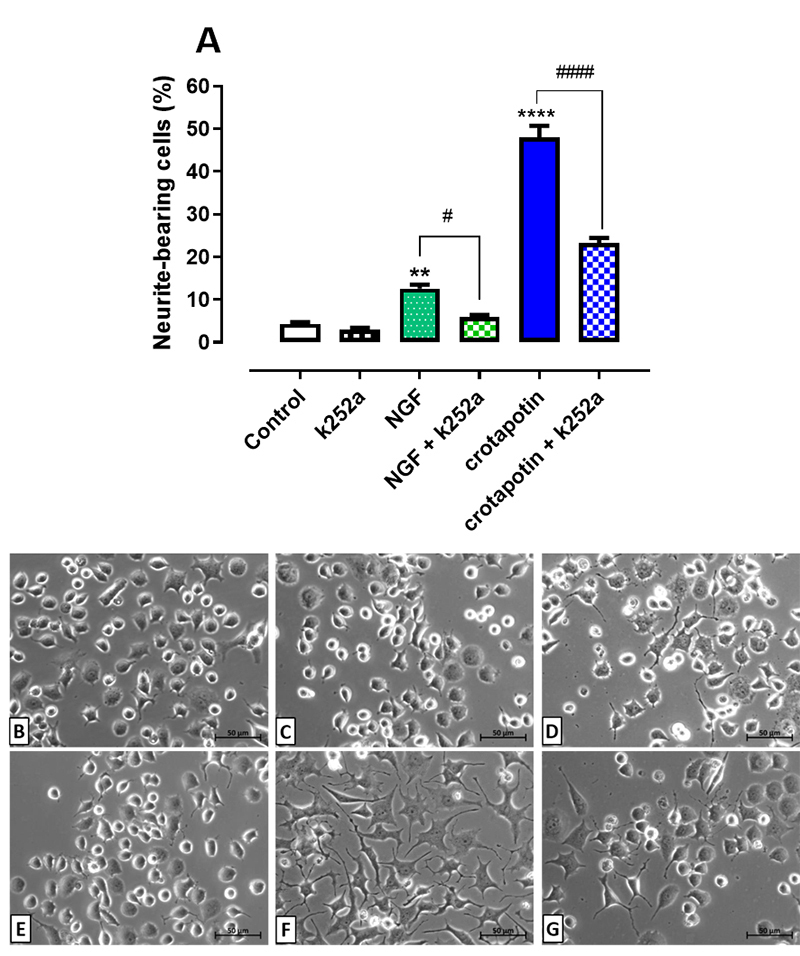
Effect of K252a (trkA antagonist) on the differentiation of PC12 cells treated with crotapotin. Cells were pretreated with K252a (100 nM) and incubated for one hour prior to the addition of NGF (100 ng/mL) or crotapotin (10 µg/mL). **(A)** The bar graph represents the mean ± SEM (n = 3). Cells with at least one neurite with a length equal to or greater than the cell body were considered differentiated and expressed as a percentage of the total cells in the field. **Significantly different from control (p < 0.01). #Significantly different from NGF (p < 0.05). ****Significantly different from control (p < 0.0001). ####Significantly different from crotapotin (p < 0.0001). **(B-G)** Inverted phase-contrast photomicrographs of **(B)** control, **(C)** K252a (100 nM), **(D)** NGF (100 ng/mL), **(E)** NGF (100 ng/mL) + K252a (100 nM), **(F)** crotapotin (10 µg/mL) and **(G)** crotapotin (10 µg/mL) + K252a (100 nM), after 72h incubation.

The NGF group (12.58 ± 0.92%, p < 0.01) and the crotapotin group (47.9 ± 2.7%, p < 0.0001) presented higher percentages of neurite-bearing cells in comparison with the control group (4.28 ± 0.46%). Pretreatment with k252a significantly reduced the neuritogenesis induced in both groups NGF+k252a (5.91 ± 0.5%, p < 0.05) and crotapotin+k252a (23.32 ± 1.1%, p < 0.0001) in relation to the groups treated solely with NGF or crotapotin, respectively. No significant difference was observed between the control group (4.28 ± 0.46%) and the group treated solely with k252a (2.94 ± 0.45%). Results are presented in Figure 2 A  -2G. 

### Inhibition of PI3K/Akt pathway reduced the differentiation induced by crotapotin

**Figure 3.**
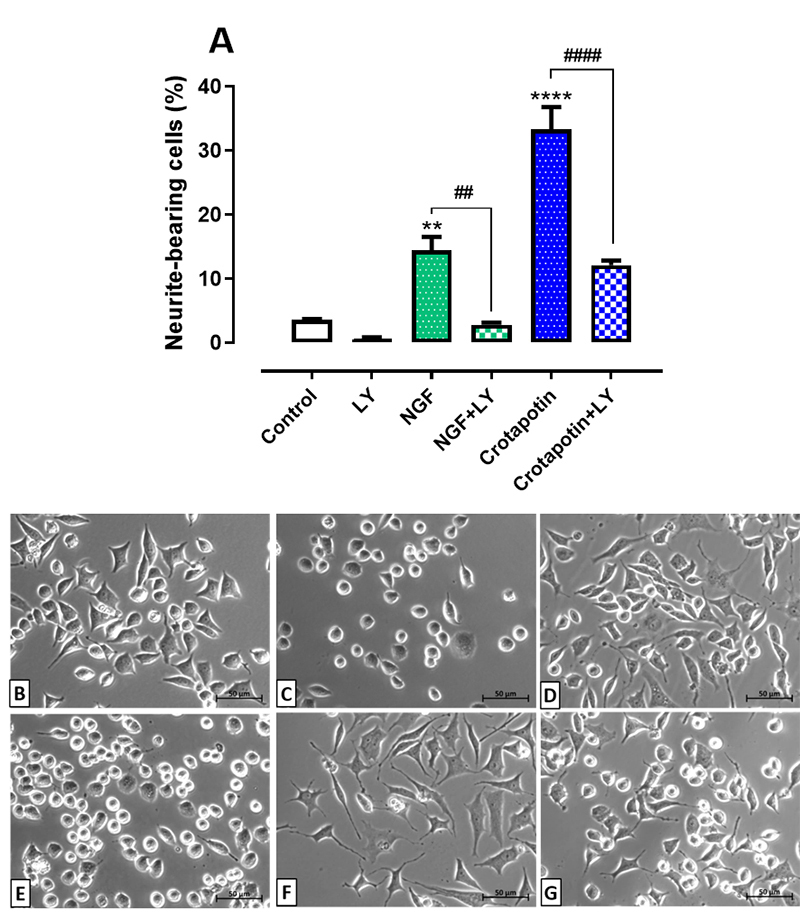
Effect of LY294002 (PI3k/Akt pathway inhibitor) on the differentiation of PC12 cells treated with crotapotin. Cells were pretreated with LY294002 (30 nM) and incubated for one hour prior to the addition of NGF (100 ng/mL) or crotapotin (10 µg/mL). **(A)** The bar graph represents the mean ± SEM (n = 3). Cells with at least one neurite with a length equal to or greater than the cell body were considered differentiated and expressed as a percentage of the total cells in the field. **Significantly different from control (p < 0.005). ****Significantly different from control (p < 0.0001). ##Significantly different from NGF (p < 0.005). #### Significantly different from crotapotin (p < 0.0001). **(B-G)** Inverted phase-contrast photomicrographs of **(B)** control, **(C)** LY294002 (30 nM), **(D)** NGF (100 ng/mL), **(E)** NGF (100 ng/mL) + LY294002 (30 nM), **(F)** crotapotin (10 µg/mL) and **(G)** crotapotin (10 µg/mL) + LY294002 (30 nM), after 72h incubation.

The NGF group (14.46 ± 2.07, p < 0.01) and the crotapotin group (33.36 ± 3.51, p < 0.0001) presented higher percentages of neurite-bearing cells in comparison with the control group (3.60 ± 0.1%). The inhibition of the PI3K/Akt signaling pathway by LY294002 (30 µM) reduced the cell differentiation induced in the groups NGF+LY294002 (2.79 ± 0.3%, p < 0.005) and crotapotin+LY294002 (12.09 ± 0.75%, p < 0.0001) in comparison with the groups treated solely with NGF or crotapotin, respectively. No significant differences were observed between the group treated solely with the inhibitor LY294002 (0.50 ± 0.33%) and the controls (3.6 ± 0.1%). Results are presented in Figure 3 A  -3G. 

### Inhibition of the MAPK/ERK pathway reduced the differentiation induced by crotapotin

**Figure 4.**
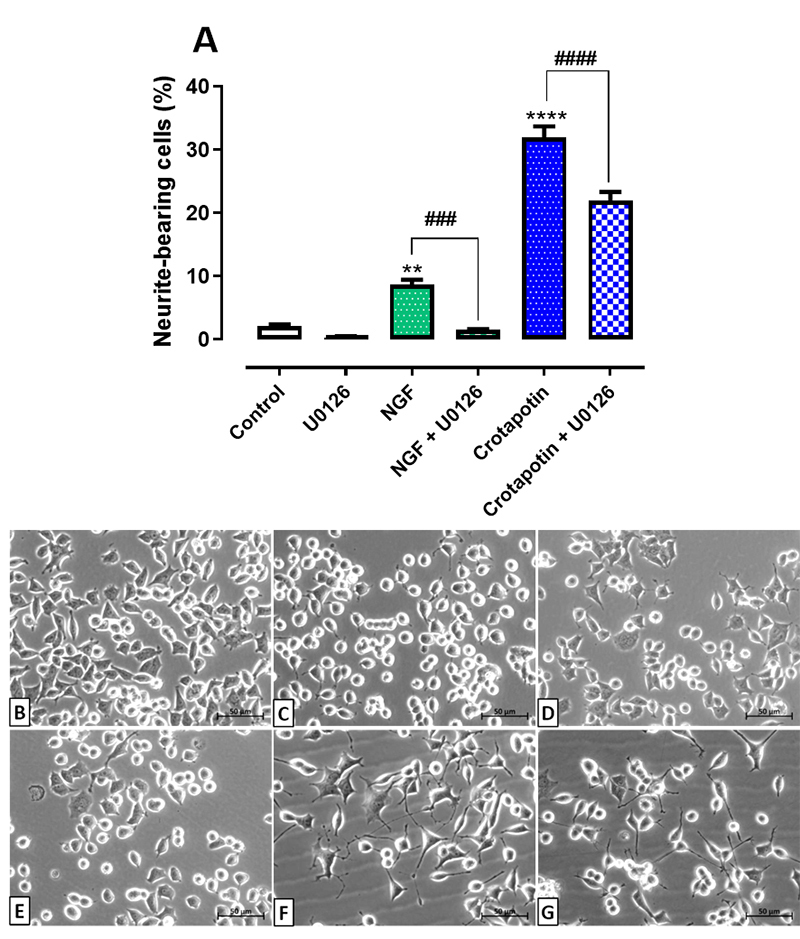
Effect of U0126 (MAPK/Erk pathway inhibitor) on the differentiation of PC12 cells treated with crotapotin. Cells were pretreated with U0126 (10 µM) and incubated for one hour prior to the addition of NGF (100 ng/mL) or crotapotin (10 µg/mL). **(A)** The bar graph represents the mean ± SEM (n = 3). Cells with at least one neurite with a length equal to or greater than the cell body were considered differentiated and expressed as a percentage of the total cells in the field. **Significantly different from control (untreated cells) (p < 0.005). ****Significantly different from control (untreated cells) (p < 0.0001). ###Significantly different from NGF (p < 0.0005). ####Significantly different from crotapotin (p < 0.0001). **(B-G)** Inverted phase-contrast photomicrographs of **(B)** control, **(C)** U0126 (10 µM), **(D)** NGF (100 ng/mL), **(E)** NGF (100 ng/mL) + U0126 (10 µM), **(F)** crotapotin (10 µg/mL) and **(G)** crotapotin (10 µg/mL) + U0126 (10 µM), after 72h incubation.

The percentage of neurite-bearing cells increased in the groups treated with crotapotin (31.93 ± 1.81%, p < 0.0001) and NGF (8.7 ± 0.78%, p < 0.005) in comparison with controls (2.09 ± 0.31). Pretreatment with U0126 reduced the neuritogenesis in the groups NGF+U0126 (1.52 ± 0.14%, p < 0.0005) and crotapotin +U0126 (21.93 ± 1.42%, p < 0.0001) in comparison with the groups treated solely with NGF or crotapotin, respectively. No significant differences were observed in the percentage of neurite-bearing cells between the group treated solely with the inhibitor U0126 (0.31 ± 0.21%) in comparison with controls (2.09 ± 0.31%). Results are presented in Figure 4 A  -4G.

### Crotapotin protected PC12 cells against the inhibition of neurite outgrowth induced by MPP^+^

**Figure 5.**
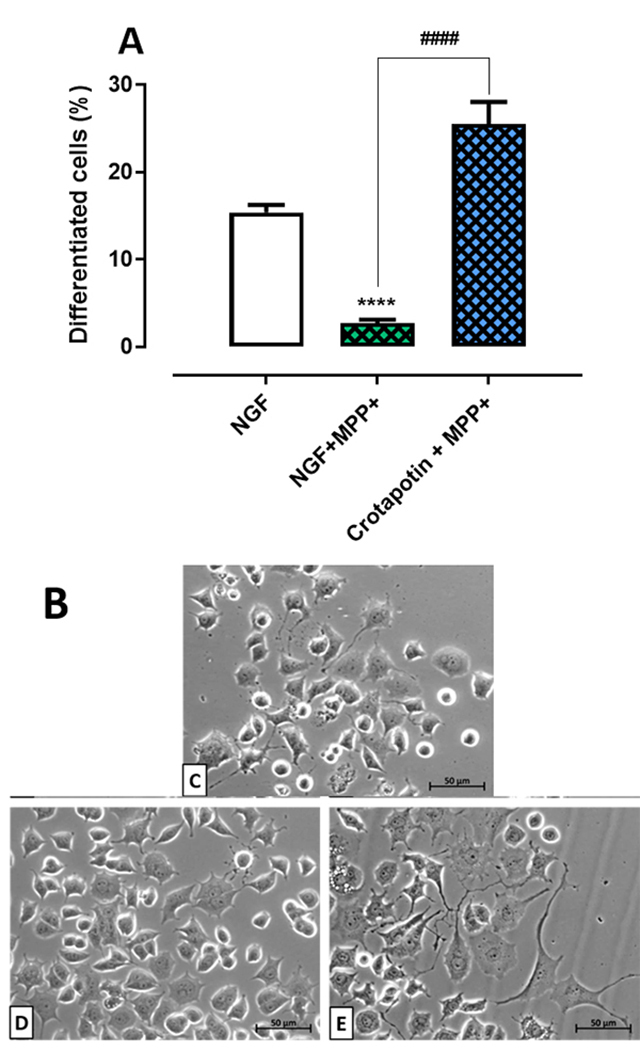
Effects of crotapotin on the differentiation of PC12 cells treated with MPP+. **(A)** The bar graph represents the mean ± SEM (n = 3). Cells with at least one neurite with a length equal to or greater than the cell body were considered differentiated and expressed as a percentage of the total cells in the field. **(B-E)** Photomicrographs of **(B)** control, **(C)** NGF (100 ng/mL), **(D)** MPP+ (100 µM), **(E)** crotapotin (10 µg/mL) + MPP+ (100 µM). ****Significantly different from control (p < 0.0001). ####Significantly different from MPP+ (p < 0.0001).

The neurotoxin MPP^+^ reduced the neurite outgrowth (2.71 ± 1.2%, p < 0.0001) in comparison with the NGF group (15.37 ± 2.57%). Crotapotin protected cells (21.68 ± 6.1%, p < 0.0001) against the inhibition of neurite outgrowth induced by MPP+ (2.71 ± 1.2%). Results are presented in Figure 5 A  -5E. 

### Crotapotin increased cell viability in MPP^+^-treated PC12 cells

**Figure 6.**
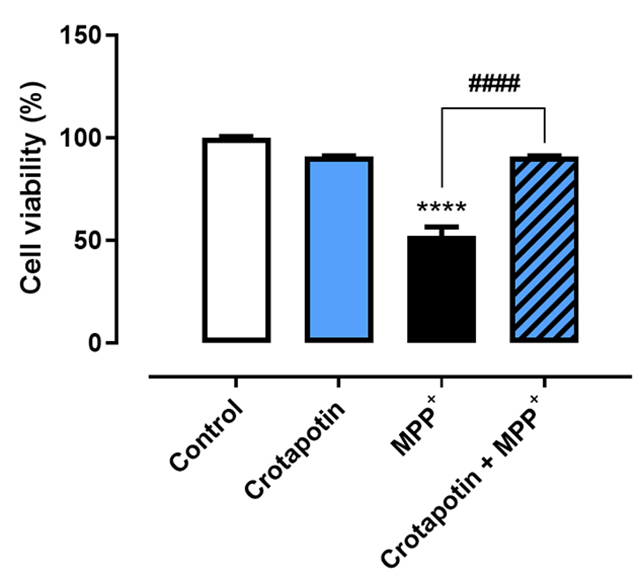
Effects of crotapotin on MPP+-induced cytotoxicity. The bar graph represents the mean ± SEM (n = 3). ****Significantly different from control (p < 0.001). ####Significantly different from MPP+ (p < 0.01).

Crotapotin (98.84 ± 4.8%) does not alter cell viability in comparison with controls (normalized to 100%). MPP^+^ significantly decreased the viability of cells (52.06 ± 4.4%, p < 0.0001) in comparison with controls. Crotapotin significantly increased the viability of cells (90.89 ± 0.30%, p < 0.0001) treated with MPP^+^ in comparison with cells treated solely with MPP^+^ (52.06 ± 4.41). Results are presented in Figure 6.

## Discussion

In this study, we have used the PC12-cell-neuronal model to evaluate the neurotrophic potential of crotapotin obtained from the venom of *Crotalus durissus collilineatus*. Additionally, we investigated the involvement of the NGF-signaling pathway in the neurotrophic mechanism of crotapotin and the protective effect of crotapotin against the toxicity of MPP+, a neurotoxin associated with Parkinsonism in animal models and humans. The PC12 cell line is a suitable model of neuronal differentiation, particularly for the investigation of compounds that mimic the NGF action, because they naturally express the NGF-selective receptor trkA, but do not express other neurotrophic receptors such as trkB or trkC, which have high affinity for BDNF and NT-3, respectively. The differentiated PC12 cells acquire the phenotype of sympathetic and dopaminergic neurons, which are affected in PD. They are electric excitable, respond to neurotransmitters, and express several neuronal markers. Additionally, they synthesize, store, release, and uptake dopamine, besides expressing α-synuclein. Therefore, PC12 cells are also a suitable model for Parkinson’s disease research [42, 57, 58]. 

Neurodegenerative diseases are characterized by the activation of multiple cellular processes such as oxidative stress, neuroinflammation, and protein aggregation, resulting in loss of neuronal function [59]. The diversity of biomolecules in animal venoms and their biotechnological potential can be useful as therapeutic tools for neuroprotection and neuromodulation. Toxins isolated from animal venoms have shown promising pharmacological and therapeutic activity [60-62] such as reducing inflammation, modulating synapses, and reducing protein aggregation [63]. 

Crotapotin (subunit A of crotoxin) is an acidic protein without enzymatic activity. The most known biological activity of crotapotin is acting as a chaperone for PLA_2_ [18] avoiding non-specific bindings of the subunit B of crotoxin [11, 64, 65]. Several isoforms of crotoxin subunits A and B have been described; they form different complexes with crotoxin and have different biological activities [11, 52, 54]. For instance, it has been demonstrated that crotoxin induces an analgesic effect and decreases motor impairment in an animal model of Multiple Sclerosis [66]; inhibits tumor growth by reprogramming macrophages and inducing antiangiogenic effect [67], and has beneficial effects on skeletal muscle repair [68]. It has also been demonstrated that crotapotin can form complexes with subunit B from *C. durissus* ssp., with PLA_2_ from other venoms, and modify the biological activity of these toxins [13, 16, 52, 69]. Accordingly, crotapotin inhibited paw edema induced in rats by PLA_2_ from *Naja naja* and *Apis mellifera* venoms, but potentiated the edematogenic effects of PLA_2_ from *Naja mocambique mocambique* venom, showing different interactions [15, 19, 20]. Cecchini and colleagues demonstrated that crotapotin inhibited the edema induced by BthTX-I, BthTX-II, PrTX-I, PrTX-III, and MjTX-II on mouse paws [70]. However, several studies have shown that crotapotin alone has different pharmacological activities. Castro and colleagues [71] evaluated the effects of crotapotin modulation on experimental autoimmune neuritis (EAN), widely used animal models of autoimmune peripheral demyelinating diseases [72, 73]. Crotapotin reduces the clinical signs and slows down the initiation of the effects associated with the disease [15, 71]. Garcia and colleagues [18] showed that crotapotin inhibited the T-cell response to Concanavalin A in a dose-dependent manner. Also, the toxin increases the production of PGE2 in T cells [18]. Oliveira et al. [56] demonstrated that the crotapotin isolated from *C. d. cascavella* venom has a bactericidal effect against *Xanthomonas axonopodis pv. passiflorae* and *Claribacteri ssp* [56]. Shimizu et al. [74] evaluated the antiviral effect of crotapotin at different stages of the Hepatitis C virus (HCV) cycle as entry, replication, and release. The authors demonstrated that treating cells with crotapotin inhibited the release of HCV in addition to interfering with lipid metabolism [74]. 

Despite all the described biological activities of crotapotin, its neurotrophic and neuroprotective effects, and the underlying mechanisms remain elusive. It is known that, in PD, there is an early stage characterized by axonal and dendritic degeneration that precedes the death of dopaminergic neurons [75-80]. Low levels of NGF and reduced trkA signaling play important roles in neurodegenerative disorders, constituting therapeutic targets in neurodegenerative disorders’ treatment [31, 81]. Depletion of neurotrophic factors such as BDNF, GDNF, and NGF has been associated with Parkinson's, Alzheimer's, and Huntington's diseases [82]. The therapeutic use of neurotrophins is limited; the clinical trials featuring the administration of NGF to treat neurodegenerative diseases have failed. The main limitations of NGF are poor bioavailability (low stability, short half-life), low blood-brain barrier permeability, and pleiotropic effects (due to the activation of the low-affinity p75 receptors, besides the high-affinity trkA receptors) [83, 84]. 

NGF is essential for the neurons’ growth, differentiation, regeneration, and maintenance [36, 85-87]. The neurotrophic signaling of NGF on trkA receptors mediates cell survival and differentiation, mainly through the activation of MAPK/Erk and PI3K/AKT pathways [88]. It has been demonstrated that, in PC12 cells, NGF activates the PI3K/Akt and MAPK/ERK pathways [89-91], promoting initiation, elongation, and branching of neurites able to form functional synapses [42, 91-97]. In this study, we demonstrated that the inhibition of one of the main modulators of these pathways (MAPK or PI3K), induced by pretreatment with specific pharmacological inhibitors (U0126, LY294002, respectively), inhibits the neurotrophic effect of crotapotin. These findings suggest that crotapotin activates the same pathways activated by the endogenous neurotrophin NGF in PC12 cells. The PC12 cell line has been largely used to explore cell differentiation and neurite outgrowth due to their well-characterized response to NGF [98, 99]. Upon NGF stimulation, PC12 cells differentiate into cells that are morphologically and functionally similar to adult sympathetic neurons; these neuron-like cells constitute a suitable model for neurobiological studies [100, 101]. NGF induces cell differentiation in PC12 cells by activating trkA receptors, which are naturally expressed by PC12 cells [102]. We observed that the neurotrophic effect of crotapotin on PC12 cells was inhibited by the trkA antagonist, k252a, which indicates that the neurotrophic mechanism of crotapotin involves the activation of the NGF-high-affinity receptor, trkA.

We further evaluated the protective potential of crotapotin against MPP^+^ toxicity. Many studies use MPP^+^ to induce damage that resembles Parkinson’s disease, in order to evaluate the effect of potential neuroprotective agents [103-106]. MPP^+^ is the active metabolite of the neurotoxin MPTP. MPP^+^ is taken up by neuronal cells through the dopamine transporter (DAT) present in the dopaminergic neurons [107]. MPP^+^ blocks complex I of the electron transport chain, inhibiting cellular respiration and ATP synthesis, therefore leading to the death of, specifically, dopaminergic neurons [107, 108]. Consistent with previous research, our results showed that MPP^+^ exposure significantly reduces PC12 cells’ viability [103] and differentiation [109-112]. Crotapotin protected PC12 cells against MPP^+^ toxicity by increasing cell viability and cell differentiation in the groups treated with MPP^+^ plus crotapotin, in comparison with the group treated with MPP^+^ alone. Several neurotrophins protect dopaminergic neurons from the toxicity of MPP^+^, including GDNF [113], NGF, BDNF, and NT-5 [114]. One of the mechanisms by which neurotrophins protect neurons is reducing oxidative stress-mediated apoptotic death through the modulation of PI3K/Akt and MAPK/Erk pathways [107, 115]. Accordingly, our study showed that crotapotin induces neuritogenesis by activating these two neurotrophic pathways, PI3K/Akt and MAPK/Erk, which might explain the neuroprotection against MPP^+^ toxicity.

## Conclusion

Taken together, our results indicate that crotapotin induces neuritogenesis in PC12 cells and protects them against MPP^+^-induced neurotoxicity. Additionally, our data suggest that the neurotrophic effects induced by crotapotin are mediated by the activation of the trkA receptor, and the downstream PI3k/Akt and MAPK/ERK pathways, which are the same cascades triggered by NGF. This is the first study to show the neurotrophic and neuroprotective potential of crotapotin. Further studies are necessary to better understand its mechanisms of action and its therapeutic potential for neurodegenerative diseases. The possible toxicity of crotapotin should be investigated in other cell models. 

### Abbreviations

MPTP: 1-methyl-4-phenyl-1,2,3,6-tetrahydropyridine; MPP+: 1-methyl-4-phenylpyridinium; Aβ: amyloid beta; TrkA: tropomyosin-related kinase A; BNDF: brain-derived neurotrophic factor; PI3K/Akt: phosphatidylinositol 3-kinase; MAPK/ERK: mitogen-activated protein kinase; NGF: nerve growth factor.; DMEM: Dulbecco’s Modified Eagle Medium; GDNF: glial cell line-derived neurotrophic factor; EDTA: ethylenediamine tetra-acetic acid; DMSO: dimethylsulfoxide; NT-5: neurotrophin 5.

## Data Availability

The datasets generated and analyzed during the current study are available from the corresponding author upon reasonable request.

## Supplementary material

The following online material is available for this article:

Additional file 1. Crotapotin analysis. **(A)** Analysis of the homogeneity of crotapotin by fast protein liquid chromatography (FPLC) with a reversed-phase C4 Jupiter column (250 × 4.6 mm, 5 μm, 300 Å, Phenomenex, Torrance, CA, USA). Mobile phase: 0.1% trifluoroacetic acid (TFA), as solution A, and 80% acetonitrile (MeCN) in 0.1% TFA, as solution B. Elution gradient: 0-100% solution B (1 mL/min). Absorbance was monitored at 280 nm. **(B)** Molecular weight analysis of crotapotin obtained by MALDI-TOF (positive linear mode) using α-cyano-4-hydroxycinnamic acid (α-CHCA) matrix. **(C)** Protein sequencing: MS/MS data were analyzed with Mascot program, against databank protein sequence deposited in the NCBI (65,519,838 sequences, 23,472,502,492 residues) and SwissProt (548,208 sequences, 195,282,524 residues). Cysteine carbamidomethylation was included as a fixed modification and oxidation of methionine was included as a variable modification. MS/MS mass tolerance was set to ± 0.8 Da.

Additional file 2. Effect of different isoforms of crotapotin on the differentiation of PC12 cells. Six crotapotin fractions (1 to 6) were evaluated for their ability to induce PC12 cell differentiation. Cells were incubated for 72h with/without NGF (100 ng/mL) or crotapotin isoforms (5 μg/mL). Data from four fields in each well were pooled and used to calculate the percentage in relation to the total number of cells in the fields. **(A)** Bar graph represents the mean ± SEM (n = 3). **(B)** Inverted contrast-phase photomicrographs of control (untreated), NGF (100 ng/mL), crotapotin fractions 1 to 6 (5 µg/mL). Cells with at least one neurite with a length equal to or greater than the cell body were counted and expressed as a percentage of total cells in the field (n = 3). ***Significantly different from control (p<0.001).
