# Supplementary material for: Fraction of C. d. collilineatus venom containing crotapotin protects PC12 cells against MPP + toxicity by activating the NGF-signaling pathway
Source: J Venom Anim Toxins Incl Trop Dis. 2024 Jun 14;30:e20230056. doi: 10.1590/1678-9199-JVATITD-2023-0056 (PMC11194915; doi:10.1590/1678-9199-JVATITD-2023-0056)
Supplement: Additional file 1. [file 1678-9199-jvatitd-30-e20230056-s1.pdf]

J Venom Anim Toxins incl Trop Dis
https://doi.org/10.1590/1678-9199-JVATITD-2023-0056

Supplementary Material to "Fraction of *C. d. collilineatus* venom containing crotapotin protects PC12 cells against MPP^+ toxicity by activating the NGF-signaling pathway"

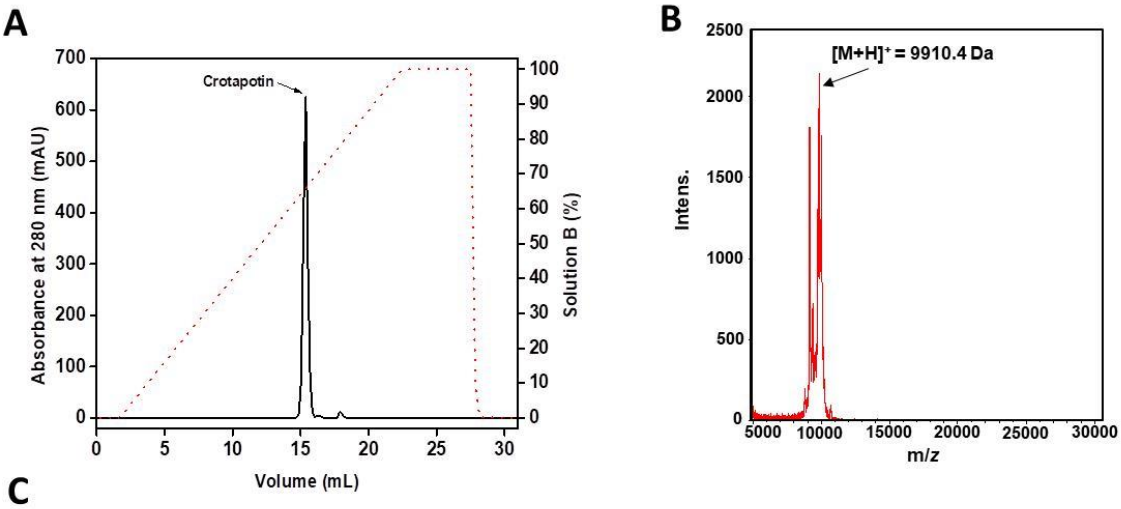

C

```
P08878        SSYGCYCGAGGQGWPQDASDRCCFEHDCCYAKLTGCDPTT    40
Crotapotin    SSYGCYCGAGGQGWPQDASDRCCFEHDCCYAKLTGCDPTT

P08878        DVYTYRQEDGEIVCGEDDPCGTQICECDKAAAICFRNSMD    80
Crotapotin    DVYTYR----------------------------------

P08878        TYDYKYLQFSPENCQGESQPC                       101
Crotapotin    ---------------------
```

Additional file 1. Crotapotin analysis. **(A)** Analysis of the homogeneity of crotapotin by fast protein liquid chromatography (FPLC) with a reversed-phase C4 Jupiter column (250 × 4.6 mm, 5 µm, 300 Å, Phenomenex, Torrance, CA, USA). Mobile phase: 0.1% trifluoroacetic acid (TFA), as solution A, and 80% acetonitrile (MeCN) in 0.1% TFA, as solution B. Elution gradient: 0-100% solution B (1 mL/min). Absorbance was monitored at 280 nm. **(B)** Molecular weight analysis of crotapotin obtained by MALDI-TOF (positive linear mode) using α-cyano-4-hydroxycinnamic acid (α-CHCA) matrix. **(C)** Protein sequencing: MS/MS data were analyzed with Mascot program, against databank protein sequence deposited in the NCBI (65,519,838 sequences, 23,472,502,492 residues) and SwissProt (548,208 sequences, 195,282,524 residues). Cysteine carbamidomethylation was included as a fixed modification and oxidation of methionine was included as a variable modification. MS/MS mass tolerance was set to ± 0.8 Da.
