# Supplementary material for: Fraction of C. d. collilineatus venom containing crotapotin protects PC12 cells against MPP + toxicity by activating the NGF-signaling pathway
Source: J Venom Anim Toxins Incl Trop Dis. 2024 Jun 14;30:e20230056. doi: 10.1590/1678-9199-JVATITD-2023-0056 (PMC11194915; doi:10.1590/1678-9199-JVATITD-2023-0056)
Supplement: Additional file 2. [file 1678-9199-jvatitd-30-e20230056-s2.pdf]

J Venom Anim Toxins incl Trop Dis
https://doi.org/10.1590/1678-9199-JVATITD-2023-0056

Supplementary Material to "Fraction of *C. d. collilineatus* venom containing crotapotin protects PC12 cells against MPP+ toxicity by activating the NGF-signaling pathway"

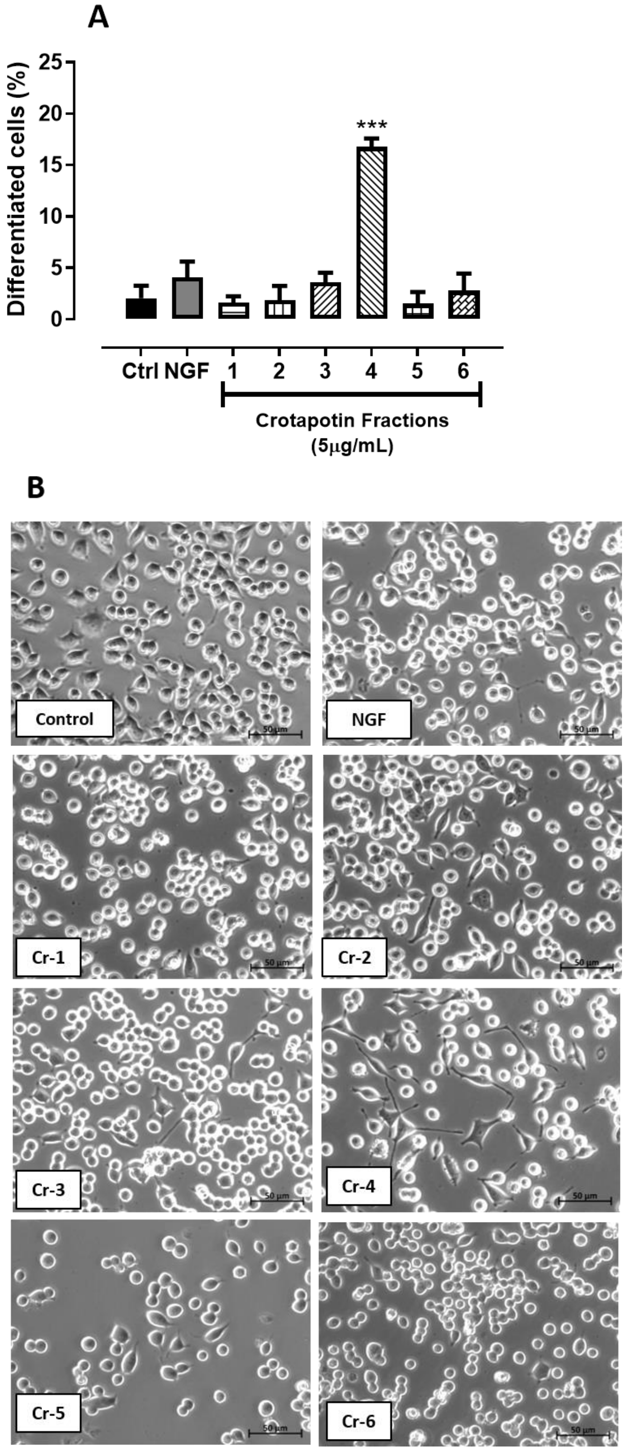

Additional file 2. Effect of different isoforms of crotapotin on the differentiation of PC12 cells. Six crotapotin fractions (1 to 6) were evaluated for their ability to induce PC12 cell differentiation. Cells were incubated for 72h with/without NGF (100 ng/mL) or crotapotin isoforms (5 µg/mL). Data from four fields in each well were pooled and used to calculate the percentage in relation to the total number of cells in the fields. **(A)** Bar graph represents the mean ± SEM (n = 3). **(B)** Inverted contrast-phase photomicrographs of control (untreated), NGF (100 ng/mL), crotapotin fractions 1 to 6 (5 µg/mL). Cells with at least one neurite with a length equal to or greater than the cell body were counted and expressed as a percentage of total cells in the field (n = 3). ***Significantly different from control (p<0.001).
